# Activity of Sodium Trimetaphosphate Nanoparticles on Cariogenic-Related Biofilms In Vitro

**DOI:** 10.3390/nano13010170

**Published:** 2022-12-30

**Authors:** Viviane de Oliveira Zequini Amarante, Alberto Carlos Botazzo Delbem, Caio Sampaio, Leonardo Antônio de Morais, Emerson Rodrigues de Camargo, Douglas Roberto Monteiro, Juliano Pelim Pessan, Thayse Yumi Hosida

**Affiliations:** 1 Department of Preventive and Restorative Dentistry, School of Dentistry, São Paulo State University (UNESP), Rua José Bonifácio, 1193, Araçatuba 16015-050, SP, Brazil; 2 Department of Chemistry, Federal University of São Carlos (UFSCar), São Carlos 13565-905, SP, Brazil; 3 Postgraduate Program in Health Sciences, University of Western São Paulo (UNOESTE), Presidente Prudente 19050-920, SP, Brazil

**Keywords:** phosphates, fluorides, nanotechnology, *Streptococcus mutans*, *Candida albicans*, dental caries, biofilms, extracellular matrix

## Abstract

In light of the promising effect of sodium trimetaphosphate nanoparticles (TMPn) on dental enamel, in addition to the scarce evidence of the effects of these nanoparticles on biofilms, this study evaluated the activity of TMPn with/without fluoride (F) on the pH, inorganic composition and extracellular matrix (ECM) components of dual-species biofilms of *Streptococcus mutans* and *Candida albicans*. The biofilms were cultivated in artificial saliva in microtiter plates and treated with solutions containing 1% or 3% conventional/microparticulate TMP (TMPm) or TMPn, with or without F. After the last treatment, the protein and carbohydrate content of the ECM was analyzed, and the pH and F, calcium (Ca), phosphorus (P), and TMP concentrations of the biofilms were determined. In another set of experiments, after the last treatment, the biofilms were exposed to a 20% sucrose solution, and their matrix composition, pH, and inorganic component contents were evaluated. 3% TMPn/F significantly reduced ECM carbohydrate and increased biofilm pH (after sucrose exposure) than other treatments. Also, it significantly increased P and F levels before sucrose exposure in comparison to 3% TMPm/F. In conclusion, 3% TMPn/F affected the biofilm ECM and pH, besides influencing inorganic biofilm composition by increasing P and F levels in the biofilm fluid.

## 1. Introduction

Dental caries consists of a non-communicable, multifactorial disease modulated by diet and mediated by biofilms, resulting from the acid released by the biofilm, leading thus to the progressive dissolution of the dental hard tissues and further tooth loss [1]. In brief, the biofilm is a very well-organized microbial community embedded in a self-produced extracellular matrix (ECM) [2]. The ECM consists of a structure composed mainly of carbohydrates, proteins, lipids, and nucleic acids, which confers to biofilm resistance, adhesivity, and a three-dimensional network that interconnects and immobilizes the cells, which makes ECM an important virulence factor for caries onset and progression [3,4]. Based on this assumption, the disruption of the ECM by affecting its components (especially proteins and carbohydrates) is considered an important target strategy for caries control [5]. 

In addition to the ECM, taking into account the caries etiology, the concentration of inorganic components such as fluoride (F), calcium (Ca), and phosphorus (P) in the oral environment has been shown to exert a crucial role in the caries process, which the higher the levels of these components in the oral environment, the lower the caries incidence, since the teeth become more prone to resist to the acid challenges [6,7,8].

Among the main microorganisms involved in the cariogenic process, *Streptococcus mutans* stands out for its ability to colonize the dental surface, produce extracellular polysaccharides from sucrose, and metabolize salivary glycoproteins [9]. *Candida albicans* has also been identified as an important microorganism in caries development and progression, acting synergistically with *S. mutans* and contributing to the maintenance of virulence factors in cariogenic biofilms [10,11,12].

Since caries is one of the most prevalent diseases worldwide, particularly in the most vulnerable population groups [13], strategies have been proposed to enhance the anticaries activity of fluoridated vehicles, such as the supplementation of such vehicles with different compounds. Among the various compounds studied, sodium trimetaphosphate (TMP) can be highlighted due to its high adsorption capacity on the dental enamel surface, acting on the selective enamel permeability, and facilitating Ca, P, and F diffusion. When adsorbed, this phosphate binds to hydroxyapatite, forming a partial barrier to acids, which retains the ions released during de-mineralization, thus aiding dental re-mineralization [14]. Moreover, F and Ca ions are retained in the biofilm as mineral deposits; when this biofilm is exposed to a cariogenic challenge, these ions can be released into the biofilm fluid and then further participate in enamel de- and re-mineralization [15,16].

Fluoride dentifrices supplemented with conventional/micro-sized TMP (TMPm) substantially affect enamel de- and re-mineralization and the inorganic composition of biofilms, in comparison to conventional dentifrices (i.e., dentifrices containing 1100 ppm F) [15,16]. Furthermore, studies evaluating the effects of TMPm, with or without F, on dual-species *S. mutans* and *C. albicans* biofilms have demonstrated that this association maintains the biofilm pH close to neutral even under highly cariogenic conditions, in addition to increasing F and P concentrations in the biofilm fluid [16]. It is noteworthy that although TMPm and F do not affect the microbial viability of such biofilms [17], TMP and F combination substantially reduces the expression of the ECM components, the biofilm’s metabolism and inorganic components of these biofilms, which play crucial roles in dental caries dynamics [17]. The effects of TMPm are further enhanced after administering this phosphate as nanoparticles (TMPn), in which TMPn combined with F increases the protective effect of fluoride vehicles against dental de-mineralization, in addition to improving their re-mineralization patterns compared to fluoride vehicles containing TMPm [14,18].

Despite the promising results on the effect of TMPm on the inorganic components of biofilms and on the biofilm ECM [16,17], information on the effects of TMPn with F is still scarce. In addition, due to the benefits of the use of TMPn over the conventional particles (i.e., TMPm) on enamel de- and re-mineralization [14,18], evaluating whether the use of TMPn and F could potentiate the effect of this phosphate on biofilms would be interesting. Therefore, in light of the crucial role of the ECM on the cariogenic process, in addition to the importance of the supersaturation of the inorganic components in the biofilm for caries control, this study aimed to evaluate the effects of TMPn, with or without F, on the pH, inorganic composition, and ECM components of mixed *S. mutans* and *C. albicans* biofilms. The null hypothesis of this study was that TMPn combined with F would not affect the analyzed biofilms.

## 2. Results

### 2.1. ECM Composition

**Figure 1:**
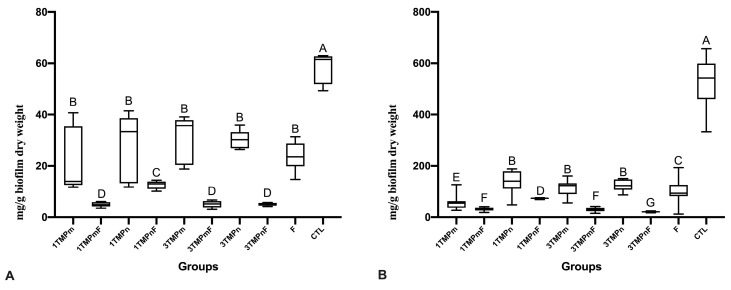
Values of protein (**A**) and carbohydrate (**B**) of the extracellular matrix of *Streptococcus mutans* and *Candida albicans* biofilms obtained after treatment with different concentrations of TMPm or TMPn, combined or not with F. Different letters denote statistical differences among the experimental groups. Bars denote the interquartile ranges. Data were submitted to Kruskal Wallis, followed by Student-Newman-Keuls’ *post hoc* test for multiple comparisons (*p* < 0.05). The experiments were performed in triplicate, in three different moments (*n* = 9). F: fluoride (1100 ppm F); TMPm: micro-sized sodium trimetaphosphate; TMPn: nano-sized sodium trimetaphosphate; CTL: negative control.

All treatments reduced the protein and carbohydrate concentrations in comparison to the CTL group (Figure 1). The groups 1TMPmF, 3TMPmF, and 3TMPnF treatments significantly reduced protein and carbohydrate concentrations more than other treatments (Figure 1). The carbohydrate concentration after 3TMPnF treatment was significantly lower than that of other treatments (Figure 1B).

### 2.2. F, Ca, P, and TMP Concentration in Biofilm Fluids

**Figure 2:**
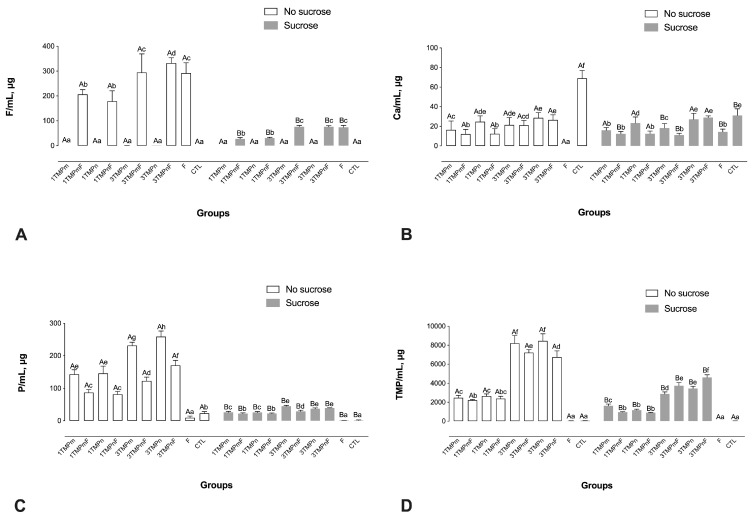
Mean values of F (**A**), Ca (**B**), P (**C**), and HMP (**D**) in the biofilm fluid, prior to and after exposure to sucrose (cariogenic challenge). Distinct upper-case letters indicate statistical within each group regarding exposure to a 20% sucrose solution (in two levels—yes or no). Distinct lower-case letters indicate statistical differences among the experimental groups (all test solutions) within each condition of sucrose exposure). Data were submitted to 2-way ANOVA, followed by Fisher LSD’s post hoc test for multiple comparisons (*p* < 0.05). Bars denote standard deviations of the means. The experiments were performed in triplicate, in three different moments (*n* = 9). Ca: calcium; F: fluoride; P: phosphorus; TMPm: micro-sized sodium trimetaphosphate; TMPn: nano-sized sodium trimetaphosphate; CTL: negative control.

The F, P, and TMP ion concentrations in the fluids of sucrose-exposed biofilms were lower than those in the fluids of biofilms without sucrose exposure (*p* < 0.001), regardless of treatment (Figure 2). The fluids of 3TMPnF-treated biofilms had a higher F concentration than the fluids of all other biofilms before sucrose exposure. However, the F levels in the fluids of 3TMPmF- and 3TMPnF-treated biofilms were similar to that of 1100 ppm F and higher than those of the fluids of 1TMPmF- and 1TMPnF-treated biofilms (*p* < 0.001) after sucrose exposure (Figure 2A).

The Ca concentrations in the fluids of 1100 ppm F-treated biofilms after sucrose exposure were significantly higher than those of biofilms without sugar exposure. However, TMPn treatment did not change Ca concentrations after sucrose exposure (Figure 1B).

The 3TMPnF-treated biofilms presented significantly higher P and TMP concentrations both prior to and after sucrose exposure (Figure 2C,D). In addition, the P concentrations were significantly higher for the biofilms treated with TMP-containing solutions without F, in comparison to their counterparts with F, both prior to and after sucrose exposure, except for the 3TMPn and 3TMPnF-treated biofilms, which were similar after sucrose exposure (Figure 2C). Also, 3% TMP presented the highest TMP concentration in the biofilm fluid in comparison to all the other groups (Figure 2D).

### 2.3. F, Ca, P, and TMP Concentration in Biofilm Biomass

**Figure 3:**
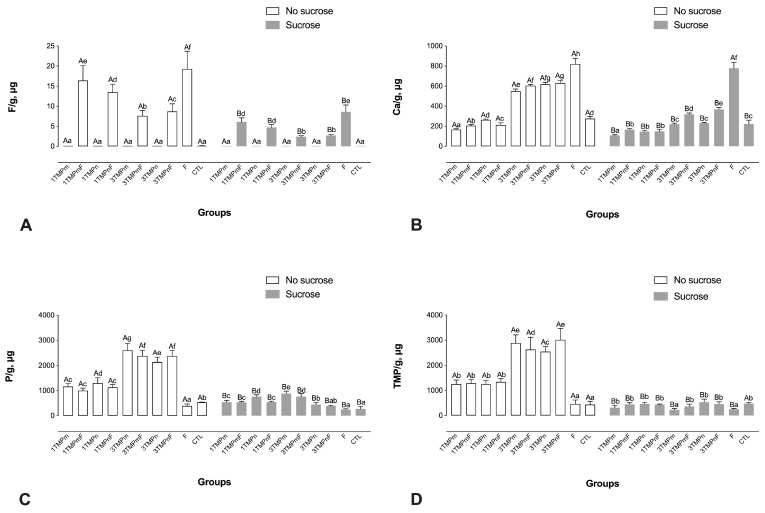
Mean values of F (**A**), Ca (**B**), P (**C**), and HMP (**D**) in the biofilm biomass, prior to and after exposure to sucrose (cariogenic challenge). Distinct upper-case letters indicate statistical differences within each group regarding exposure to a 20% sucrose solution (in two levels—yes or no). Distinct lower-case letters indicate statistical differences among the experimental groups (all test solutions) within each condition of sucrose exposure). Data were submitted to 2-way ANOVA, followed by Fisher LSD’s post hoc test for multiple comparisons (*p* < 0.05). Bars denote standard deviations of the means. The experiments were performed in triplicate, in three different moments (*n* = 9). Ca: calcium; F: fluoride; P: phosphorus; TMPm: micro-sized sodium trimetaphosphate; TMPn: nano-sized sodium trimetaphosphate; CTL: negative control.

F, P, and TMP concentrations in the biomass of sucrose-exposed biofilms were lower after the cariogenic challenge than those in the biomass of biofilms without sucrose exposure (*p* < 0.001), regardless of treatment (Figure 3). Moreover, only the biomass of F-treated biofilms had significantly higher F ion concentrations than that of all other biofilms before and after sucrose exposure (Figure 3A). In addition, the biomass of 1TMPmF- and 1TMPnF-treated biofilms showed significantly higher F concentrations than the 3TMPmF- and 3TMPnF-treated biofilms before and after sucrose exposure (Figure 3A).

The biomass of F-treated biofilms had a significantly higher Ca ion concentration than that of other biofilms before and after sucrose exposure (Figure 3B). In addition, the biomass of biofilms treated with 3TMPm and 3TMPn, with and without F, had higher Ca concentrations than the biomass of those treated with 1TMPm and 1TMPn, with or without F (Figure 3B).

TMP presented the highest P values, regardless of particle size or the presence of F, before sucrose exposure (Figure 3C). The TMP levels in the biomass were proportional to those observed in the treatment solutions, in which 3TMPnF promoted a greater amount of TMP in the biomass than the other treatments before sucrose exposure (Figure 3D).

### 2.4. pH Measurement

**Figure 4:**
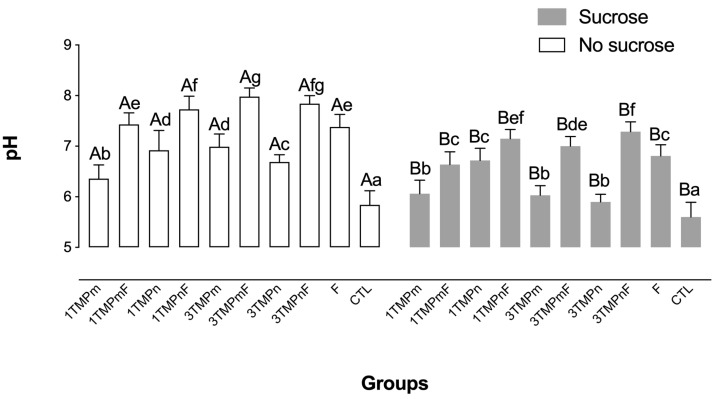
Mean pH values prior to and after exposure to sucrose (cariogenic challenge). Distinct upper-case letters indicate statistical differences within each group regarding exposure to a 20% sucrose solution (in two levels—yes or no). Distinct lower-case letters indicate statistical differences among the experimental groups (all test solutions) within each condition of sucrose exposure). Data were submitted to 2-way ANOVA, followed by Fisher’s LSD *post hoc* test for multiple comparisons (*p* < 0.05). Bars denote standard deviations of the means. The experiments were performed in triplicate, in three different moments (*n* = 9). TMPm: micro-sized sodium trimetaphosphate; TMPn: nano-sized sodium trimetaphosphate; CTL: negative control.

The pH of biofilms after the cariogenic challenge was lower than that of biofilms not exposed to sucrose (*p* < 0.001; Figure 4). Regardless of the concentration, the biofilms treated with TMPn, combined with F, presented significantly higher pH than that of biofilms treated only with F (i.e., 1100 ppm F) and their micro-sized counterparts before and after sucrose exposure (Figure 4). 

## 3. Discussion

Recent studies have observed that combined TMP and F treatment substantially affect the pH, ECM composition, and inorganic components of *S. mutans* and *C. albicans* mixed biofilms [16,17]. Furthermore, reducing the particle size to the nanometer scale (i.e., TMPn) improves its effectiveness [14,18]. This study evaluated the effects of TMPn with or without 1100 ppm F on the inorganic composition, pH, and ECM components of *S. mutans* and *C. albicans* mixed biofilms, demonstrating that the nanoparticles increased the F concentration in the biofilm fluid, promoted pH maintenance close to a neutral level, and significantly decreased the carbohydrate and protein concentrations in the biofilm ECM than its microparticulate counterpart or the solution containing only 1100 ppm F, thus rejecting the null hypothesis of the study.

The production of an ECM containing water-insoluble glucans is an important virulence factor associated with dental caries pathogenesis [19,20,21], which is the reason why such variables were analyzed in the present study. Therefore, the reduction of protein and carbohydrate concentrations observed in this study is interesting, as it may influence by reducing cell adhesion and aggregation, biofilm resistance, and acid niche formation in biofilms [19]. The ECM of 3TMPnF-treated biofilms was substantially reduced in terms of protein and carbohydrate concentrations, which may reflect the TMP and F concentration, mainly because of the particle size, as these smaller particles have a high proportion of surface area in relation to volume, as well as a high percentage of atoms on the surface than larger particles, which increases their reactivity [22,23]. These results corroborate those of in situ studies, which showed that the combined treatment of 1100 ppm F and 3% TMPm or TMPn substantially reduced carbohydrate in comparison to 1100 ppm F without TMP [18]. In line with these trends, similar data were observed for another phosphate salt (i.e., sodium hexametaphosphate) using the same biofilm model, which showed that the reduction of the particle size to nanoscale also enhanced the effects of the phosphate on the biofilm ECM components [24]. In addition, in line with the above-mentioned trends, disaggregated hydroxyapatite nanoparticles demonstrated increased inhibitory effects on the metabolism and acidogenicity of oral biofilms formed in vitro in comparison to their micrometric counterpart [25].

The action of TMP in reducing carbohydrates in the ECM may be partially related to the increased Ca availability in the biofilm [26]; however, the *S. mutans* and *C. albicans* mixed biofilms with high Ca concentrations (CTL for biofilm fluid and F for biofilm biomass) did not demonstrate the greatest reductions in the matrix components evaluated. Although the reasons for this pattern are not fully understood, some factors may have influenced the results. The evaluations performed in the present work were conducted in vitro in microtiter 6-well plates without the presence of a dental substrate for biofilm formation and continuous salivary flow, which might have influenced the pattern of the ionic composition of the biofilms assessed. Also, as nanometric TMP particles present a high percentage of atoms on the surface, increase reactivity [14,18,22,23] with the biofilm, and possibly improve interaction with the Ca ions present in the culture medium and the structures of microorganisms (i.e., cell wall and outer membrane), so that these ions might not be available for quantification in the biofilm fluid or biomass [26,27].

High TMP concentrations in the biofilm fluid can interfere with the pH, as TMP is also used as a buffering agent [28]. The buffering capacity of TMP associated with F with respect to the pH drop has already been reported in a previous study, which reported that 1% TMPm associated with 500 ppm F maintains the pH of biofilms close to neutral, even after cariogenic challenge [16]. These results are related to the buffering capacity of this phosphate due to its chelating property; accordingly, the presence of TMP in the biofilm fluid causes the binding of TMP to H^+^ and Na to OH^−^ [28]. 

Interestingly, the reduction in the TMP particle size led to an additive effect on the pH of the biofilms as even after sucrose exposure, the biofilms treated with 1TMPn and 3TMPn, combined with F, had significantly higher pH than those treated with their microparticulate counterparts and the positive control (1100 ppm F). In fact, such data are in line with those in literature for another phosphate, which demonstrated that the reduction of sodium hexametaphosphate particle size enhanced its buffering capacity [29]. Such a trend can also be justified by the higher reactivity of nanoparticles over the microparticles, leading to the enhancement of the effects observed for the groups treated with the conventional particles [22,23].

With respect to the inorganic composition of biofilms, this study demonstrated that the F and P concentrations were reduced in the biofilms after sucrose exposure, as observed in situ [30] and in vivo [31]. Almost all biofilm minerals (fluid and biomass) were reduced after a cariogenic challenge [32]. 3TMPnF treatment promoted F concentration in the biofilm fluid higher than other treatments with the same F concentration (1100 ppm), but reduced ionic P and P from TMP concentration than treatment with its counterpart without F, possibly because TMP is considered an inorganic polyphosphate and therefore a metal chelator [33]. Therefore, biofilm cations, such as Ca, may induce the binding of TMP to bacterial cells [34,35], decreasing the number of F ions binding with the bacteria, thus reducing and increasing their levels in the biofilm biomass and fluid, respectively, showing that TMP particle size can potentiate this interaction.

The high availability of P in the fluids of TMP-treated biofilms may also interfere with CaHPO_4_^0^ formation, which is of extreme importance for enamel re-mineralization due to its higher diffusion coefficient in enamel than that of ionic Ca [36]. The combination of TMP and high concentration F in the biofilm fluid would release more CaF^+^ in the oral environment, reacting with HPO_4_^2–^ and forming CaHPO_4_^0^ and HF^0^, elements with higher diffusion coefficients in subsurface caries lesions than that of ionic Ca [36], which can affect mineral dynamics and enamel de-mineralization.

In line with previous data, which demonstrated that F concentrations in the environment are dependent on Ca concentrations [37], treatments with 1100 ppm F led to significantly higher Ca concentrations in the biomass, and the Ca and F concentrations were proportional. For bivalent cations (e.g., Ca), the binding to the microbial wall becomes monovalent in the presence of F once this ion competes with anionic macromolecular groups. This promotes the binding of F to the bacteria via the Ca bridge (Ca-F) and increases the negative sites in the bacteria, which favors the cation binding to the bacterial surface [35].

The limited number of microorganisms used, the absence of a dental substrate for biofilm formation, and the lack of continuous salivary flow found in clinical conditions limit the protocol used in this study. Nonetheless, these limitations became necessary to minimize possible interference and not induce the results. Therefore, taking into account the influence that these aspects exert on caries dynamics [38,39,40], developing new studies using other experimental models would be interesting.

## 4. Materials and Methods

### 4.1. Details on the Processing and Characterization of the TMP Nanoparticles 

The processing and characterization of the TMP nanoparticles were performed as described by Danelon et al. [14]. In brief, 70 g of pure (micrometric) sodium trimetaphosphate (Na_3_O_9_P_3_, Sigma Aldrich [St. Louis, MO, USA] purity ≥ 95% CAS 7785-84-4) was ball milled with zirconia spheres (2 mm diameter) in 1 L of isopropanol. After 48 h, the resulting powder was separated from the alcoholic media and ground in a mortar. The powder crystallinity was characterized by X-ray diffraction (XRD) using a Rigaku Dmax 2500 PC (Austin, TX, USA) diffractometer, by which it was observed that the milling processing reduced the particle size of the TMP powders without affecting their crystalline structure. Also, it was noted a pattern of the broader peaks due to the smaller crystallites for the TMP after 48 h ball milling, estimating an average particle size of 22.7 nm. In addition, scanning electron microscopy (SEM) images were obtained using Philips XL-30 FEG (Amsterdam, the Netherlands), showing the particles agglomerated before the milling [14]. 

### 4.2. Microorganisms and Growth Conditions

American Type Culture Collection (ATCC) reference strains were used: *S. mutans* ATCC 25175 and *C. albicans* ATCC 10231. *C. albicans* cultures were seeded in Sabouraud dextrose agar (ASD Difco [Le Pont de Claix, France]) and cultivated for 24 h at 37 °C, while cultures of *S. mutans* were seeded in brain heart infusion (BHI Agar; Difco [Le Pont de Claix, France]) and incubated in 5% CO_2_ at 37 °C for 24 h. After the cultivation of the ASD plates, 10 mL were suspended in Sabouraud dextrose broth (Difco [Le Pont de Claix, France]) and incubated at 37 °C overnight, under shaking at 120 rpm for *C. albicans*. For *S. mutans*, a loop was suspended in 10 mL of BHI broth (Difco) and incubated statically overnight in 5% CO_2_ at 37 °C [16].

After incubation, the microorganisms were recovered by centrifugation (8000 rpm, 5 min at 15 °C), and pellets were formed, which were washed twice with 10 mL of saline solution (0.85% NaCl). Subsequently, the cells had their concentration adjusted: the fungal cells at 10^7^ cells/mL, through a Neubauer chamber and an optical microscope, and the bacterial cells through the spectrophotometer at 640 nm to a concentration of 10^8^ cells/mL in artificial saliva [16]. The artificial saliva used was supplemented with sucrose and thus had the following composition for 1 L of deionized water: 2 g of yeast extract (Sigma-Aldrich, St Louis, MO, USA), 5 g of bacteriological peptone (Sigma-Aldrich), 4 g of sucrose (Sigma-Aldrich), 1 g of mucin (Sigma-Aldrich), 0.35 g NaCl (Sigma-Aldrich), 0.2 g CaCl_2_ (Sigma-Aldrich), and 0.2 g KCl (Sigma-Aldrich). The pH of the saliva was adjusted with NaOH to 6.8 [8]. For the preparation of biofilms, suspensions of 4 mL (1 × 10^7^ cells/mL *C. albicans* + 1 × 10^8^ cells/mL *S. mutans*) were made in artificial saliva, and then they were inserted into the wells of microtiter 6-well plates and incubated at 37 °C for 72 h. The artificial saliva renewal was done with 2 mL every 24 h [16].

### 4.3. Treatment of the Biofilms

The biofilms formed for 72 h were treated a total of three times (72, 78, and 96 h) for 1 min [16]. The treatment solutions used were: 1% TMPm (1TMPm), 3% TMPm (3TMPm), 1% TMP + 1100 ppm F (1TMPmF), 3% TMPm + 1100 ppm F (3TMPmF), 1% TMPn (1TMPn), 3% TMPn (3TMPn), 1% TMPn + 1100 ppm F (1TMPnF) and 3% TMPn + 1100 ppm F (3TMPnF) [6,7,8,10]. For the positive control group, 1100 ppm F (as NaF) was used, and the negative control was artificial saliva (CTL). 

### 4.4. Analysis of the Composition of the Extracellular Matrix of Biofilms

Biofilms were formed as described above in 6-well plates containing 4 mL of the inoculum. After the last treatment with the solutions, the biofilms were washed with NaCl (0.85%) and scraped from the bottom of the wells using cell scrapers (Kasvi [São José dos Pinhais, Brazil]) and resuspended in NaCl (0.85%). Subsequently, the biofilm samples were sonicated for 30 s at 30 W, and the suspensions obtained were vortexed for 2 min. Finally, the suspensions were centrifuged at 3000× *g* for 10 min, and the supernatant was filtered using a nitrocellulose filter (0.22 μm; Orange Scientific [Braine-l’Alleud, Belgium]) [41]. This supernatant was used for the analysis of proteins and carbohydrates.

For the determination of the biofilm dry weight, the sample of the scraped biofilms was filtered through a cellulose acetate membrane (0.22 μm), and the membrane was dried at 60 °C until the dry weight was constant [17]. The membrane was also weighed before biofilm filtration. Therefore, the final dry weight of the biofilms was the difference between the two measurements. 

The protein content of the extracellular matrix of the biofilms was determined by the bicinchoninic acid method (BCA Kit, Sigma-Aldrich [St. Louis, MO, USA]), using bovine serum albumin as a standard. For this, a volume of 25 μL of the obtained supernatant was transferred to wells of 96-well plates containing 200 μL of the mixture of reagents A and B from the BCA kit. The plates were incubated at 37 °C for 30 min, and the absorbance of the obtained solutions was read at 562 nm [17,41]. Carbohydrate was quantified as described by Dubois et al. [42] using glucose as a standard. 500 μL of the supernatant was collected and deposited in glass tubes, and to the supernatant was added 500 μL of 9% phenol and 2.5 mL of sulfuric acid (95–98%; Sigma-Aldrich). The obtained solution was homogenized and kept at rest for 15 min at room temperature, after which the absorbance was read at 490 nm.

### 4.5. pH Measurement

The pH was determined using a pH electrode (PHR-146 Micro Combination pH Electrode; Fisher Scientific), which was previously calibrated with pH 7.0 and 4.0 standards. At another time of the experiments, after the last treatment, the biofilm was exposed to a 20% sucrose solution for three min simulating a cariogenic challenge. The sucrose was then removed, and the biofilms scraped off and transferred to microtubes (after 1 min of sucrose removal), allowing pH determination [16].

### 4.6. Analysis of F, Ca, P and TMP in the Biofilm Fluid

After transferring the biofilm to the microtubes, they were centrifuged (15,267× *g*) at 4 °C for 5 min, and the biofilm fluid was separated and collected [16]. F analysis was performed using an ion-selective (Orion 9409 BN; Thermo Scientific [Waltham, MA, USA]) and reference electrodes (Orion 900100; Thermo Scientific [Waltham, MA, USA]), both coupled to a potentiometer (Orion; Thermo Scientific [Waltham, MA, USA]). Calibration curves for fluid F analysis were made using standards of 0.09, 0.18, 0.36, 0.72, and 1.44 μg F/mL (for biofilms treated with solutions without F) and 6.25, 12.5, 25, 50, and 100 μg F/mL (for biofilms treated with F-containing solutions). The total ionic strength adjusting buffer (TISAB II) was added, which was administered under the same conditions as the samples in a 1:1 ratio [16].

Ca ion was measured by spectrophotometry in a plate reader (EON Spectrophotometer; EON, Biotek, Winooski, VT, USA) at a wavelength of 650 nm, adapting the method described by Vogel et al. [43], in which Arsenazo III was used. An aliquot of 5 μL in duplicate for both standards and samples was added with 50 μL of Arsenazo III and 50 μL of deionized water. Subsequently, they were shaken for 60 s in the microplate reader, promoting the reaction between the sample and Arsenazo III before obtaining the resulting absorbance.

Phosphorus ion and phosphorus from TMP were measured according to the method by Fiske and Subbarow [44]. Whereas, for samples that were exposed to cariogenic challenge, determination of P from TMP was done by boiling water bath process at 60 °C for 6 h [7].

### 4.7. Analysis of F, Ca, P and TMP in the Biofilm Biomass

To measure the inorganic composition of the biofilm biomass, 0.5 mol/L of HCL was added to the microtubes containing the transferred biofilms at a rate of 0.5 mL/10.0 mg of wet plate weight [45] and then it was homogenized. The resulting combination was kept for 3 h at room temperature under constant stirring (120 rpm) and then centrifuged (11,000× *g*) for 1 min [46]. 400 μL of the sample was removed, and the same volume of 0.5 mol/L NaOH was added.

F ion was measured as previously described, using standards containing 0.09, 0.18, 0.36, 0.72, and 1.44 μg F/mL (for biofilms treated with solutions without F) and 0.8, 1.6, 3.2, 6.4, and 12.8 μg F/mL (for biofilms treated with solutions containing F). For biofilms exposed to cariogenic challenge, the calibration curve was performed using standards of 0.2, 0.4, 0.8, 1.6, and 3.2 μg F/mL [16]. Ca and P were determined as described above for biofilm fluid.

### 4.8. Statistical Analyzes

Data on the protein and carbohydrate of the ECM passed the normality test (Shapiro–Wilk) and were submitted to 1-way ANOVA, followed by the Student-Newman-Keuls test. Data on the pH and the inorganic components of the biofilms passed the normality test and were submitted to 2-way ANOVA, followed by Fisher LSD’s test. Statistical analyzes were performed using the SigmaPlot 12.0 (Systat Software Inc., San Jose, CA, EUA) software, adopting *p* < 0.05. All experiments were performed in triplicate, in three different moments (*n* = 9).

## 5. Conclusions

Given the context discussed above, it can be concluded that 3% TMPn combined with F led to significant reductions in the biofilm ECM carbohydrate content and increases in the biofilm pH (after sucrose exposure) in comparison to its microparticulate/conventional counterpart (i.e., 3% TMP) combined with F, or the 1100 ppm F solution (the positive control). In addition, this combination affected the inorganic components of the biofilms, increasing the P and F availability in the biofilm fluid before the sucrose exposure. The data presented in this study contribute to the understanding of how TMPn and F act on the dynamics of dental caries, elucidating the activity of this combination on cariogenic-related biofilms.

## Data Availability

The data presented in this study are available on request from the corresponding author.
